# Body Composition and Its Perception among Professional Female Volleyball Players and Fitness Athletes (Silesia, Poland)

**DOI:** 10.3390/ijerph191911891

**Published:** 2022-09-20

**Authors:** Agnieszka Białek-Dratwa, Wiktoria Staśkiewicz, Mateusz Grajek, Aleksandra Filip, Mateusz Rozmiarek, Karolina Krupa-Kotara, Oskar Kowalski

**Affiliations:** 1 Department of Human Nutrition, Department of Dietetics, Faculty of Health Sciences in Bytom, Medical University of Silesia in Katowice, Jordana 19, 41-808 Zabrze, Poland; 2 Department of Food Technology and Quality Evaluation, Department of Dietetics, Faculty of Health Sciences in Bytom, Medical University of Silesia in Katowice, Jordana 19, 41-808 Zabrze, Poland; 3 Department of Public Health, Department of Public Health Policy, Faculty of Health Sciences in Bytom, Medical University of Silesia in Katowice, Piekarska 18, 41-902 Bytom, Poland; 4 Department of Sports Tourism, Faculty of Physical Culture Sciences, Poznan University of Physical Education, Królowej Jadwigi 27/39, 61-871 Poznań, Poland; 5 Department of Epidemiology, Department of Epidemiology and Biostatistics, Faculty of Health Sciences in Bytom, Medical University of Silesia in Katowice, Piekarska 18, 41-902 Bytom, Poland

**Keywords:** body composition, body image perceptions, female, athletes

## Abstract

Female athletes experience both sociocultural and sport-specific pressures of an ideal body and appearance and are vulnerable to dissatisfaction with their bodies. Among sport-specific pressures, the type of sport is a predictor of body image dissatisfaction. The study included 150 females: 50 volleyball players, 50 bodybuilding and fitness athletes, and 50 female students, who were the control group. Body composition and perception and evaluation of one’s own body were assessed. BMI was similar in the study group of female athletes and the control group, but the bodybuilding and fitness athletes had the lowest body fat, while the control group had the highest. Perception of one’s own body in the aspect of the evaluation of specific body parts was highest among bodybuilding and fitness athletes, while in the aspect of body condition, the best results were obtained by volleyball players. Most female volleyball players were dissatisfied with their current body weight, as were women in the control group, in contrast to female bodybuilding and fitness athletes, who were most often satisfied with their current body weight.

## 1. Introduction

In sport and exercise psychology research, body image has received much attention and has been recognized as an important factor associated with physical activity and sports behavior [1]. The definition of body image states that “it is a multidimensional construct focusing on both the appearance and functioning of the body” [1]. The perceptual dimension is how a person perceives and describes their appearance and body functioning, while the cognitive dimension assesses thoughts about body appearance and functioning [1,2]. Body image has become a popular topic over the past 40 years [3]. Interest in this area coincides with growing public health concerns about weight status, physical inactivity, obesity, eating disorders, and the associated spectrum of health consequences [4]. In a CBOS (Center for Public Opinion Research) survey of 880 adult Poles, women were twice as likely as men to be critical of their appearance (34% vs. 18%) [5].

Female athletes experience both sociocultural and sport-specific pressures to have an ideal body and appearance and are, therefore, at risk for body dissatisfaction [6,7]. Body dissatisfaction includes negative thoughts and feelings about one’s own body and the perceived discrepancy between one’s current and ‘ideal’ body. A person’s perception of his or her body can change depending on the context in which he or she functions [8]. Elite athletes have reported having both athletic and social body image [9]. Thus, body image can be measured in the context of both sports and everyday life [10].

Among sport-specific pressures, the type of sport is a predictor of body image dissatisfaction [11]. In a review of studies on body image in athletes and non-athletes, Varnes et al. indicated that playing sports prevented negative perceptions of one’s body image, but this protection was less in women and elite athletes [12]. Analogous results were reported by Kong and Harris, in which female athletes such as dancers and gymnasts reported higher levels of body dissatisfaction than athletes involved in sports, i.e., ball sports, regardless of the level of participation [13]. In addition, professional athletes reported higher levels of body dissatisfaction than those involved in recreational sports [14].

Dissatisfaction with one’s body shape and physical appearance, along with the desire to be thinner to improve athletic performance, can lead to the development of eating disorders [15]. Individuals who are dissatisfied with their bodies may engage in unhealthy behaviors such as restricting dietary intake and using ergogenic or laxative drugs, and as a result, this may contribute to the development of eating disorders [16].

In our study, we focused on the self-perception of body image in professional female athletes of different disciplines, because, according to the available literature, it seems that the phenomenon of body dissatisfaction with different types of physical activity is still under-researched. In addition, research results often vary depending on the type of sport, level of competition, age, and research methodology [17]. Bodybuilding and fitness bodybuilding competitions involve assessing the body aesthetics and physique of female athletes. For this reason, female athletes pay very close attention to their appearance, which may determine the evaluation of their body image and result in a more critical perception of their figure. Volleyball, on the other hand, as a team sport, is characterized by a different type of effort and a different system of evaluating athletic form. The players are focused on scoring as many points as possible, and their body is not subject to aesthetic evaluation; instead, attention is paid to the correct composition of the body mass that determines exercise capacity and fitness in general.

From a health perspective, satisfaction with one’s body is one of the primary factors in preventing unhealthy weight-related behaviors, especially in female athletes. In addition, assessing self-perceptions of one’s body can be used as a tool to screen for and predict the possibility of eating disorders, especially among female athletes. These disorders can have serious health consequences [18].

The purpose of this study was to assess body mass composition and evaluate perceptions of body image among women engaged in various types of physical activity (volleyball players, bodybuilding and fitness athletes, and non-sport women). The essence of the study was to answer the question of whether body composition and its perceptions are represented by the female members of the sports studied, because, as noted above, a disturbed body image can be an important trigger for further health-related problems in sports people.

In the study, the research hypothesis was that women regardless of anthropometric parameters (body weight, body fat content, muscle mass content, visceral fat content) perceive their parameters to be worse than they are regardless of the type of sport and lack of physical activity. Two completely different sports were included in the study: one in which, by definition, very precise control of anthropometric parameters is required (bikini fitness athletes), volleyball players, and a control group of women who do not participate in sports at the level of competition.

It was assumed that body composition and its perceptions are more important (pay more attention to it) for women engaged in bodybuilding and fitness than for women playing volleyball.

## 2. Materials and Methods

### 2.1. Study Design

The study was conducted between March and June 2021. Data were collected by a direct survey, using a questionnaire technique. The study was conducted by the guidelines of the Declaration of Helsinki, while all procedures were approved by the Bioethics Committee of the Silesian Medical University in Katowice (PCN/0022/KB/68/I/20). All participants consented to participate in the study.

### 2.2. Participants

The study included 112 adult women professionally practicing sports between the ages of 19 and 29 and were training in the Silesian agglomeration. Both groups of athletes were studied during a macro-cycle of training.

The criteria for inclusion in the study were defined as being 18 years of age or older, had provided consent to participate in the study, the athletes had a membership in a sports association at the time of the study, and could speak the Polish language to the extent that the study could be carried out, as well as the absence of an injury that excluded the athlete from the representation of a sports club for more than 31 days in total during at least 1 year from the time of the study. Participants who were absent from at least one element of the survey were excluded from the study group.

The study included a control group, which consisted of 50 female students from the Silesian University of Technology aged 19–23. The inclusion criterion for the control group was defined as being at least 18 years of age, had consented to participate in the study, and were not a member of a sports association for at least 5 years from the time of the study. The exclusion criterion for the control group was systematic physical activity, defined as physical activity occurring at least 3 times a week for 45 min each.

The study included 112 female athletes, however, taking into account the inclusion and exclusion criteria, 100 women were finally qualified for the study: 50 women belonging to the Polish Volleyball Federation (PZPS), and 50 women belonging to the Polish Bodybuilding, Fitness and Triathlon Sports Association (PZKFiTS). It was decided that such sports that represent completely different criteria in the space of body composition and body image would be selected. This procedure allowed for the emergence of intergroup comparisons and the reference of results to a control group in the form of people with low or standard physical activity.

The study was conducted based on Polish law, i.e., the Act on Medical and Dental Professions of 5 December 1996, which includes a definition of medical experimentation. Study participants gave written informed consent to participate in this study. All study participants were informed of the purpose of the study, its anonymity, and were asked to accept the data sharing policy. Information about informed and voluntary participation in the study was included at the beginning of the survey. The study was conducted by the Declaration of Helsinki.

### 2.3. Procedures

#### 2.3.1. BMI

Height (cm) and body mass (kg) were measured to the nearest 0.1 cm and 0.1 kg (SECA 756, Seca gmbh & co. kg, Hamburg, Deutschland), respectively, with the subject wearing underwear and no shoes. BMI was calculated as body mass (kg) divided by height (m) squared. The results were used to assess height–weight ratios against standards for the European population and WHO recommendations [19].

#### 2.3.2. Diet Quality

The quality of the respondents’ diets was determined using the DQI (Diet Quality Index) [20], which takes into account eight parameters: the proportion of energy from total fat and saturated fatty acids in the diet, the intake of fruits and vegetables, products that are sources of complex carbohydrates, and the supply of protein, calcium, sodium, and cholesterol. Points are awarded for each parameter evaluated (0 reflected implementation of dietary recommendations, and 2 reflected non-compliance). Depending on the number of points, the quality of the daily rations of the respondents was defined as: unsatisfactory (11–16 pts.), acceptable (8–10 pts.), moderate (6–7 pts.), good (4–5 pts.) or high (0–3 pts.).

#### 2.3.3. Body Composition

Body composition was assessed using a multi-frequency electrical bioimpedance analyzer (BIA) (TANITA MC-780 P MA, Tanita Corp., Tokyo, Japan).

The Tanita MC-780 body composition analyzer using a tetrapolar eight-point tactile electrode system was used, which separately measures the impedance of the subject’s trunk, arms, and legs at three different frequencies (5 kHz, 50 kHz, 250 kHz). The analyzer allows to obtain the full body composition of the subject in about 20 s. The device operates using a current of 90 uA.

The tests were performed according to a standard protocol by the device manufacturer’s recommendations. The subjects fasted, did not consume alcohol or caffeine at least 24 h before the test, the measurement was performed at a fixed time, after defecation, at least 24 h after the end of intense physical activity, without shoes or socks, in underwear, with clean and dried feet and hands without applied cream and lotion.

Before each testing session, the analyzer was checked with a calibration circuit of known impedance (resistance = 500.0 Ω; reactance = 0.1 Ω; 0.9% error). Information concerning the subject (age, gender, and height) was entered by the experimenter. Participants had to step on the foot electrodes barefoot and maintain an evenly distributed weight on the measurement platform while holding a pair of electrodes fixed on the display unit. Then, participants extended their arms in front of the chest, maintaining a steady position until measurements were completed. Body fat and skeletal muscle mass (expressed as a percentage of total body mass) were predicted using the manufacturer’s valid and reliable equations. The visceral fat index was determined by a program recommended by the analyzer manufacturer.

As a result of the body mass composition analysis, the following parameters were assessed: percentage of body fat (BF) [%], fat-free mass (FFM) [kg], total body water (TBW) [kg], bone mass (BM) [kg], level of visceral fat.

#### 2.3.4. Body Image Perception

The following questionnaires were used to assess perceptions and evaluate body image: ‘The Body Esteem Scale—BES in Polish adaptation’ [21], ‘Confidence related to one’s appearance [22], and the ‘35-item Contingencies of Self-Worth Scale’ part related to appearance [23].

The BES allowed us to determine the subjects’ attitudes toward their bodies. In this study, we used the female version of the scale. Responses to the BES scale were given using a 5-point Likert scale: 1—I have strong negative feelings, 2—I have moderately negative feelings, 3—I have no feelings, 4—I have moderately positive feelings, and 5—I have strong positive feelings. The BES scale for women consisted of three subscales: sexual attractiveness, weight control, and physical condition. In our study, we assessed two aspects: ‘physical fitness’ and ‘weight control.’ The ‘Weight Control’ aspect took into account those parts of the body whose appearance can be changed through exercise or dieting. The ‘Physical Conditioning’ aspect refers to the evaluation of parameters such as endurance, strength, and agility. In the ‘Body Conditioning’ aspect, physical endurance, reflexes, muscle strength, energy level, arms, physical coordination, agility, health, and physical conditions were evaluated. On the other hand, the ‘weight control’ aspect evaluated appetite, waist, thighs, physique, buttocks, hips, legs, figure, abdomen, and body weight. The maximum score in ‘physical fitness’ was 45 points (5 points in 9 aspects). In ‘weight control,’ the maximum number of points was 50 points (5 points in 10 aspects). Interpretation for the BES was based on norms for women aged 20–29, due to the age of the study group of women.

The second scale of opinion regarding one’s own body was based on two scales: ‘Confidence related to one’s appearance’ and the ‘35-item Contingencies of Self-Worth Scale’ part related to appearance. The combined scales included responses on a 5-point Likert scale: 1- doesn’t fit me, 2—doesn’t fit me, 3—neither fits nor doesn’t fit me, 4—fits me, 5—fits me. The higher the score, the more positive the respondent was about their body image. Sample questions are described in Table 1.

In addition, the survey included data that included questions about the length of training experience, the number of training units per week, cooperation with a nutritionist, the use of diets, and the number of meals consumed.

The questionnaire was completed by the respondents during individual consultations to conduct the survey fairly and meticulously.

#### 2.3.5. Statistical Analysis

Statistical analyses were performed using Statistica v.13.3 (Stat Soft Poland) and the R package v. 4.0.0 (2020) under the GNU GPL (The R Foundation for Statistical Computing).

To present quantitative data, mean values and standard deviations were calculated. For qualitative data, percentage notation was used. Qualitative data were expressed as numerical values determined by mathematical methods to make statistical inferences. Compliance with the normal distribution was checked using the Shapiro–Wilk test. For distributions deviating from the normal distribution, their conformity was checked using the Mann–Whitney U test or the Kruskal–Wallis test. Pearson’s χ2 test was used to assess the relationship. Measures of relationship strength such as Kendall’s Tau B and C coefficients were used to measure the strength of the relationship. A one-way ANOVA test to assess BMI was also conducted. A value of *p* < 0.05 was used as a criterion for statistical significance.

## 3. Results

### 3.1. Characteristics of the Study Group

The complete study material was obtained for 150 females: 33.3% (*n* = 50) professional volleyball athletes, 33.3% (*n* = 50) elite bodybuilding and fitness athletes, and 33.3% (*n* = 50) physically inactive female students from the Silesian University of Technology.

A dietitian’s advice was used by 14.0% (*n* = 22) of the total subjects, including 16.0% (*n* = 8) of volleyball players, 22.0% (*n* = 11) of bodybuilding and fitness athletes, and 6.0% (*n* = 3) of the control group. There was a significant statistical difference between the groups regarding the use of diets, with 20.0% (*n* = 10) of female volleyball players and 58.0% (*n* = 29) of female bodybuilding and fitness athletes declaring the use of diets, while no one in the control group gave such a response.

Dietary supplements were used by 36.4% (*n* = 55) of the women surveyed. A statistically significant variation was shown between the analyzed groups. Female volleyball players were the most frequent users of supplements (*n* = 27; 54.0%), half of the female bodybuilding and fitness athletes surveyed also declared the use of dietary supplements, while the control group used supplementation by a mere 6.0% (*n* = 3).

**Figure 1:**
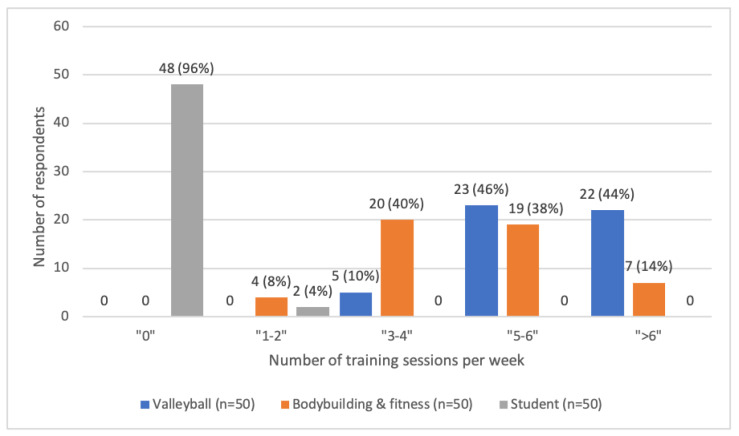
Quantities of declared training units per week in the studied groups.

An analysis of the number of declared training units per week is shown in Figure 1.

### 3.2. Physical Characteristics and Body Composition

The age, physical characteristics (height, body mass, and BMI), body composition variables (fat content (PB%), lean body mass (LBM), and total body water (TBW)) about sport discipline are shown in Table 2.

The mean age of the study group was 22.6 ± 2.7 years. Considering height, the highest was the volleyball players—a result of the nature of their sport, while the bodybuilding and fitness athletes were lower, and the control group was the lowest. The volleyball players had the highest average body weight, the control group was lighter, and the bodybuilding and fitness athletes were the lightest. In the study, BMI was calculated based on body weight and height. The bodybuilding and fitness athletes had the lowest mean BMI, and the volleyball players and the control group have similar values. Bodybuilding and fitness athletes had the lowest body fat. Considering lean body mass, female bodybuilding and fitness athletes had higher muscle mass than the other study groups, including the control group. The groups differed in all the characteristics described (*p* < 0.05).

### 3.3. Assessment of Diet Quality Using the DQI

**Figure 2:**
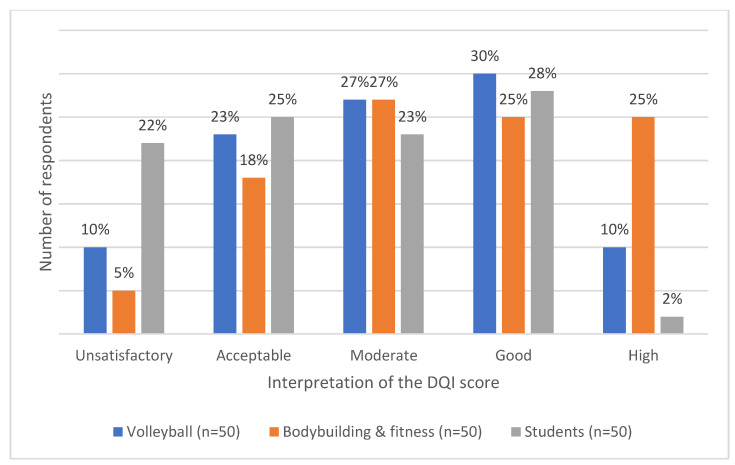
Interpretation of the DQI score.

Based on the evaluation of the respondents’ menus using the DQI, it was estimated that the best quality of rations was characterized by those belonging to the bodybuilding and fitness group—a high quality according to the DQI among 25% of the respondents. As for the good quality of the menus, the most points were given to those in the volleyball group—30% of cases. The control group (students) (22%) had a worse score than the other groups—T = 13.4002; *p* = 0.0001 – Figure 2.

### 3.4. Results of Self-Perception Based on the Body Assessment Scale

Based on the BES Body Evaluation Scale, among the female volleyball players studied, the average score obtained (29.4 points) in the aspect of the evaluation of individual body parts was lower than among bodybuilding and fitness athletes (32.1 points). The lowest average was obtained by the control group (27.4 points). Considering the physical condition of the body, the volleyball players obtained the highest average (29.9 points), and a slightly lower average was obtained by the bodybuilding and fitness athletes (29.7 points). A significantly lower average value was obtained by the control group (26.3 points). A statistically significant relationship was found that women in the bodybuilding and fitness group were more likely to indicate dissatisfaction with their body shape (T = 12.8791; *p* = 0.0022). The information is presented in Table 3.

### 3.5. Results of Self-Perception in the Subjective Evaluation of the Surveyed Women

Evaluation of body fitness based on the BES Body Evaluation Scale showed that the differences between the study groups were in physical fitness (T = 13.2211; *p* = 0.0001), muscle strength (T = 12.9844; *p* = 0.0008), and physical conditioning (T = 11.1212; *p* = 0.0066), where the control group had the lowest scores. The evaluation of individual body parts showed differences between thighs (T = 10.2397; *p* = 0.0186), physique (14.7832; *p* = 0.0063), buttocks (T = 11.2683; *p* = 0.0008), hips (T = 11.1145; *p* = 0.0003), legs (T = 13.0901; *p* = 0.0186), and condition (11.3221; *p* = 0.0066), where the control group also tended to score lowest, except for hip physique, where the volleyball players scored lowest. Table 4 shows the detailed results of the self-image assessment of the female subjects.

Assessment of self-esteem and self-confidence related to one’s body appearance showed differences between the study groups. Female bodybuilding and fitness athletes scored lower on the questions ‘It bothers me that I don’t look better (T = 11.2911; *p* = 0.0213), ‘My self-esteem doesn’t depend on whether I look better or worse (T = 12.9981; *p* = 0.0001), ‘I wish I could change my physical appearance’ (T = 10.8882; *p* = 0.0015) and ‘I would have much more success on dates if I looked better’ (T = 12.8731; *p* = 0.0002). In contrast, the women in the control group scored higher than the athletes on the question ‘Most people would probably consider me physically unattractive’ (T = 10.3356; *p* = 0.0041). More often than not, the control group had worse self-esteem about their looks than the female bodybuilding and fitness athletes (T = 10.3121; *p* = 0.0007). At the same time, female volleyball players also had worse self-esteem about their appearance than female bodybuilding and fitness athletes (Table 5).

The subjective directions of the desire to change body shape among the subjects were statistically significantly different. Female volleyball athletes wanted to reduce body fat the most (50.0%; *n* = 25), as did female bodybuilding and fitness athletes (24.0%; *n* = 12). In the control group, the largest number of female respondents answered that they wanted to decrease body fat (40.0%; *n* = 20) and decrease body fat while increasing muscle (40.0%; *n* = 20). A statistically significant difference was shown in the aspect of fat reduction and muscle mass gain. Those in the control group were more likely to show this need (T = 13.4732; *p* = 0.0075). The exact data are shown in Table 6.

**Figure 3:**
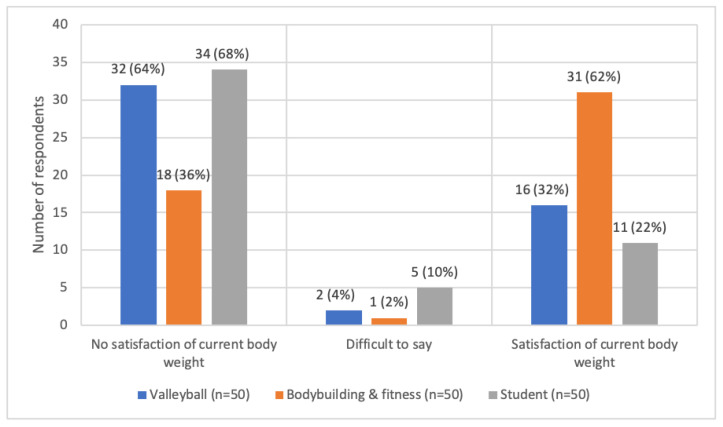
Satisfaction with their current body weight in the study groups.

The majority of female volleyball players are not satisfied with their current body weight (64.0%; n= 32), as are women in the control group (68.0%; *n* = 34). In contrast, 32.0% of female volleyball players (*n* = 16) and 22.0% (*n* = 11) of women in the control group are satisfied with their body weight. This is in contrast to female bodybuilding and fitness athletes, where 36.0% (*n* = 18) said they were not satisfied, and 62.0% (*n* = 31) were satisfied with their current body weight. The above was proven to be statistically dependent—T = 13.2337; *p* = 0.0002 (Figure 3).

**Figure 4:**
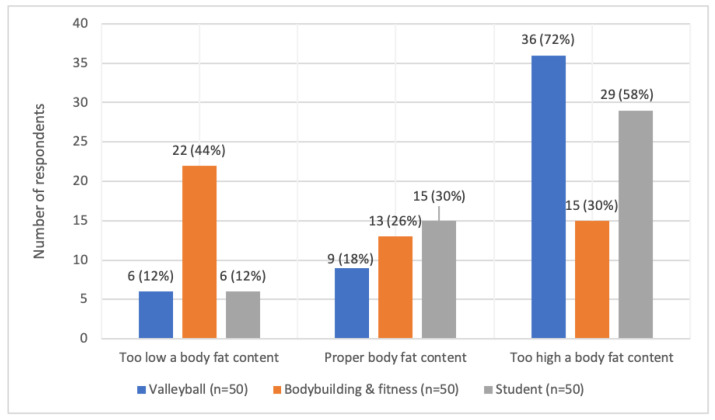
Subjective assessment of the propriety of body fat in the study groups.

Subjective assessment of the propriety of having body fat varied. The largest number of female volleyball players (72.0%; *n* = 36) felt that they had too much body fat, similar feelings were held by women in the control group (58.0%; *n* = 29), while only 30.0% (*n* = 15) of bodybuilding and fitness athletes gave this response. Only 18.0% (*n* = 9) of female volleyball players, 26.0% (*n* = 13) of female bodybuilding and fitness athletes, and 30.0% (*n* = 15) of women in the control group felt they had normal body fat. A total of 12.0% (*n* = 6) of volleyball players and women in the control group and as many as 44.0% of bodybuilding and fitness athletes believed their body fat content was too low. The above was proven to be statistically dependent– T = 14.7211; *p* = 0.0001 (Figure 4).

## 4. Discussion

The perception of one’s own body in the aspect of the evaluation of individual body parts was highest among bodybuilding and fitness athletes, while in the aspect of physical fitness, the best results were obtained by female volleyball players. The subjective directions of the desire to change body shape among the female participants varied: half of the volleyball players wanted to reduce body fat, while the control group wanted to reduce body fat while increasing muscle mass. Women involved in bodybuilding and fitness wanted to change their physique the least. Most of the volleyball players were dissatisfied with their current body weight, as were the women in the control group, in contrast to the bodybuilding and fitness athletes, who were most often satisfied with their current body weight. Considering the results obtained, women in both the bikini fitness athletes, the volleyball players, and the control group had an inappropriate perception of their bodies. In all the groups studied, their body perception was worse than reality. It is worth noting that even in the bikini fitness group, this was the case. The literature [1,2,3,4,5,6,7,8,9,10,11,12,13,14,15,16,17,18,19,21,22,23,24,25,26,27,28,29,30,31,32,33,34,35] emphasizes that the belief in the beauty of one’s body influences a person’s psychological and social functioning, and first and foremost, their attitude to themselves and their relationships with other people. As outlined in the introduction, this seems to be particularly important for women, and certainly among women who exercise intensively, who, to achieve a certain sporting result, must take care of their bodies and push them with various forms of exercise. The current study shows that factors such as BMI level and body composition are important determinants in the perception of one’s own body. Interestingly, the results differ by study group. Women representing more silhouetted disciplines (in this case, bodybuilding and fitness) pay more attention to their appearance and rate it worse than women from team sports (such as volleyball). The results of studies by other researchers are presented below and compared with the achievements of the current study.

The author’s results did not evaluate the impact of the psycho-emotional state on self-perception, but evaluated two sports groups and compared them with a control group. Based on this investigation, it was concluded that self-image depends, among other things, on the sport represented. Nonetheless, such relationships are evident in the literature. Zarek [25] emphasizes that satisfaction with one’s body (positive body image) is associated with higher self-esteem, a sense of attractiveness, greater self-confidence, and a sense of personal happiness. The author surveyed 177 people aged 19–53 (including 148 women and 29 men) and used the Body Image Questionnaire to measure body image. The lack of gender differences in the evaluation of one’s own body was surprising to the researchers. Rybicka-Klimczuk and Brytek-Matera [26] believe that the most frequently mentioned emotions that women feel about their body image are feelings of satisfaction or dissatisfaction with their external appearance and fear of fat gain. The authors included a group of 130 women in their study. They measured body image variables using the Silhouette Test, the Body Dissatisfaction Scale, and the Fear of Physical Appearance Scale, while behavioral aspects of eating disorders were examined using the Attitudes Toward Eating Test. It turned out that the studied adolescent girls and women between the ages of 20 and 25 revealed the strongest tendencies to engage in eating disorder behaviors. The body mass index of the women studied was within the norm, only the group between 40 and 50 years of age was overweight. Daubenmier [33] compared body satisfaction, body-awareness (body-awareness), body responsiveness (body-awareness), and level of self-objectification (self-objectification) in women practicing yoga and attending aerobics classes. The researcher found that more positive body image occurred in women practicing yoga than in those attending aerobics classes, regardless of body weight (measured by BMI value). The pre-subject literature [34] emphasizes that the relationship between body image and physical activity is complex and that physically active people respond more strongly to new stressful situations and are more sensitive to new stimuli. Garst [35] studied 64 middle-aged women, who were divided into an experimental group (mean age 39.7 years) and a control group (mean age 40.1 years) with 32 subjects in each group. The study groups did not differ in terms of age, education level, or nature of work. At the outset, physical fitness was determined in all women and the level of satisfaction with the perception of their body image was measured; then, group I of women undertook systematic recreational gymnastics, and group II carried out normal life activities. It turned out that the recreational gymnastics practiced by the women had a favorable effect on their global assessment of self-image perception, as well as on the assessment of health, muscularity, chin, voice, shoulder width, knees, eyes, and energy level.

The author’s results showed a difference in attitudes toward self-perception between the control group and the athletes, but also between female athletes in different sports. Women engaged in bodybuilding and fitness were more satisfied with their appearance than volleyball players and women in the control group, which may be related to the different types of exercise which shape the figure differently. Likewise, a study on athletes’ self-esteem was conducted by Esenturki et al. who, using the Rosenberg Self-Esteem Scale, measured the level of self-esteem in 120 students at Gazi University Physical Education and Sports Academy who regularly engage in physical activity. In the results presented, they found that regardless of age or gender, all athletes had high levels of self-esteem [27]. Another team of researchers, who also used the Rosenberg Self-Esteem Scale in their study, were Gencer and Ilhan [28]. The authors selected badminton training athletes from 12 sports clubs in Turkey as the study group. These athletes were also characterized by high levels of self-esteem. The score achieved was not related to either the length of training, gender, or the age of the athletes [28].

The authors’ results also indicate that people characterized by higher levels of physical activity are more satisfied with their physiques than inactive people, but the level of satisfaction is also influenced by the type of sport practiced. Similarly, a study by Ziolkowska and Dobrogoszcz analyzed body image and the tendency of bigorexia in strength-trained women [29]. The results showed no difference between the type of training and body image, in contrast to their results [29]. In contrast, Prichard and Tiggermann’s study found that cardio-type training significantly correlates with a negative evaluation of one’s body image, as opposed to strength activity. It seems reasonable to conclude that any physical activity has many important functions in human development, but it is those who exercise intensively, especially strength training, who have better self-esteem and a positive self-image [30]. A study by Ferrari et al. analyzed satisfaction with one’s self-image among university students in Brazil, and it was shown that up to 70% of them were not satisfied with their appearance [31]. Radwan et al. conducted a study with students from a university in the United Arab Emirates; they found that as many as 78.9% of students wanted to increase or decrease their body weight [31]. In their study, as many as 68% of female students were not satisfied with their body weight. An important issue was raised in a study conducted by Kruger et al. in the United States. The authors showed that those who were dissatisfied with their weight showed lower levels of physical exercise compared to those who were satisfied with themselves [23].

The benefits of women’s participation in sports are extensive; women’s sports and physical activity can result in improved self-esteem and body image, higher academic success, and higher bone mineral density compared to inactive women [36]. However, in 1992, a correlation was found between eating disorders, lack of menstruation, and osteoporosis in athletes, termed the female athlete triad (FAT). In 1997, the American College of Sports Medicine (ACSM) established a position statement on the triad; at first, symptoms accompanying eating disorders, lack of amenorrhea, and osteoporosis were required to define the disorder. Many women, due to restrictive criteria, were not successfully identified, so it was discovered that the symptoms of the triad occur along a spectrum, rather than as “absolute” symptoms [37,38]. Therefore, an updated position statement was published in 2007, using the terms low energy availability (with or without nutritional disorders), menstrual disorders, and low bone mineral density [36]. The characteristics of the triad of female athletes are closely related to reduced energy intake, often caused by a deformed body image, social pressures regarding thinness, and sport-specific pressures, e.g., low body fat in female athletes of figure sports. It has been found that athletes in weight-dependent aesthetic sports are more likely to use restrictive weight control methods than athletes in other sports [39]. Recently, a pressure for a slim body for athletes has increased, and as a result, female athletes perceive more pressure than non-athletes about their body image [40]. The promoted notion that reducing body weight or body fat can improve athletic performance has increased the risk of developing eating disorders and the triad of female athletes in recent times [40]. It should be noted here that in recent years, attention has been increasingly drawn to the RED-S phenomenon among professional athletes. RED-S syndrome refers to a relative energy deficiency in sports [41]. Low energy availability can lead to several physiological disorders and negatively affect athletic performance [42]. The syndrome applies to both men and women, compared to the triad of sportswomen, which applied only to women [43]. A high risk of deficiency occurs when the energy supply is less than 30 kcal/kg of lean body mass. Moderate risk occurs when the energy supply is between 30–45 kcal/kg of lean body mass. An adequate energy supply is maintained when an athlete supplies the body with about 45 kcal/kg of lean body mass [44]. In men, these values may be lower, although there is a lack of relevant research on the subject [45]. For example, according to a study conducted in New Zealand on a group of fitness club attendees whose moderate weekly exercise was 150 min, exactly 33.5% of participants were classified as at risk for low energy availability. Among females, the percentage was significantly higher [46]. Dipla et al. [46] point out that chronic energy deficiency (weeks to years) can result in reduced training adaptations and an increased risk of injury [47]. RED-Scan can lead to health problems, which include menstrual disorders (in women); disorders of the immune, digestive, cardiovascular, hematological, metabolic, and endocrine systems; a disruption to the process of growth and development of the body; a decrease in the rate of metabolism; disorders in the mental sphere; a decrease in bone mineral density [48]. Unfortunately, RED-S is still not widespread in the sports community. This results in a higher risk of its occurrence. A study conducted in the USA found that among 931 physicians, only 37.0% had heard of RED-S syndrome [49].

In addition, it is worth noting that in recent years, the subject of much social debate has been so-called body positivity, i.e., coming to terms with the shape of one’s body in favor of a better mental condition. As outlined above, inadequate perceptions of one’s own body can be an inflammatory factor in many subsequent afflictions, to which those involved in aesthetic, strength, and team sports may be particularly vulnerable. Misperception of the body and the constant noticing of its inadequacies in the context of its shape is a symptom of cognitive disorders that can lead to the development of eating, mood, and anxiety disorders, while constant weight control leads to malnutrition, weakened organs and systems, increased risk of injury, and even death. Given this, the results presented in the paper and their conclusions can serve as a monitoring of the phenomenon, since the data that were collected in the study concerns the first symptoms of abnormalities that may signal a more serious problem.

## 5. Strengths and Limitations

Undoubtedly, an important asset of the study is the fact that it is pioneering in Poland. There is little research of this type in Poland, and it is important because sports and physical activity play an important role in people’s lives. In addition, life comfort and well-being defined by physical appearance is the number one topic in general and on social media. In addition, the size of the study group and the use of standardized diagnostic tools deserve appreciation.

The study, of course, is not without limitations. First of all, it should be noted that the groups were different from each other; therefore, comparisons cannot be generalized, but in further studies, we will make an effort to assess body composition and its perception in other groups of athletes. The current study undertook a probe of comparisons between different sports to build a background for further research projects that are in the authors’ plans.

## 6. Conclusions

In the study group of female athletes and the control group, BMI was comparable, but body composition parameters such as body fat content and muscle mass differed between the study groups and the control group. Female bodybuilding and fitness athletes had the lowest body fat content, while the control group had the highest—this is understandable given the need to control body fat content among bikini fitness athletes. Considering lean body mass, the female bodybuilding and fitness athletes had a higher muscle mass content than the other groups studied, including the control group. The diet quality of the female bikini fitness athletes was better than in the other groups. Nevertheless, it was inappropriate for more than half of the athletes.

## Figures and Tables

**Table 1:** Questions on the opinion scale about one’s own body.

1. It disturbs me that I don’t look better.	1	2	3	4	5
2. My self-esteem does not depend on whether I look better or worse.	1	2	3	4	5
3. I am satisfied with my appearance.	1	2	3	4	5
4. My self-esteem is affected by how attractive I find my face and its appearance	1	2	3	4	5
5. I look better than the average person	1	2	3	4	5
6. My self-esteem suffers whenever I think I don’t look good.	1	2	3	4	5
7. I am happy that I look so good	1	2	3	4	5
8. My self-esteem is not related to how I feel about my appearance	1	2	3	4	5
9. Most people will probably consider me physically unattractive.	1	2	3	4	5
10. When I think I look attractive, I feel better about myself.	1	2	3	4	5
11. I would like the power to change my physical appearance.	1	2	3	4	5
12. I would have had much more success on dates if I looked better.	1	2	3	4	5

**Table 2:** Characteristics of the study group and body composition analysis.

	Control Group N = 50 X ± SD Min–Max	Volleyball N = 50 X ± SD Min–Max	Bodybuilding and Fitness N = 50 X ± SD Min–Max	Total N = 150 X ± SD Min–Max	*p*-Value
Age (years)	20.2 ± 1.2 19.0–23.0	23.9 ± 2.9 19.0–29.0	23.7 ± 1.8 20.0–27.0	22.6 ± 2.7 19.0–29.0	**0.0001**
Body height (cm):	164.2 ± 5.7 151.0–175.0	178.1 ± 6.4 165.0–198.0	167.0 ± 5.2 156.0–177.0	169.7 ± 8.3 151.0–198.0	**0.0001**
Body mass (kg)	59.1 ± 10.9 47.0–96.1	69.8 ± 7.4 58.7–93.2	58.2 ± 9.2 47.0–83.7	62.3 ± 10.6 47.0–96.1	**0.0001**
BMI (kg/m^2^)	21.9 ± 6.2 18.8–25.0	21.9 ± 4.4 19.8–24.1	20.9 ± 2.4 19.7–22.1	21.7 ± 4.8 18.8–25.0	**0.0001**
Percentage of body fat (%)	24.6 ± 5.7 13.9–42.9	20.2 ± 3.5 13.3–27.3	17.2 ± 5.0 9.5–29.4	20.2 ± 33.8 9.5–42.9	**0.0001**
Lean body mass [(kg)	55.1 ± 4.1 45.2–61.5	55.4 ± 4.2 47.2–65.1	58.4 ± 5.3 45.7–69.3	56.3 ± 4.8 45.2–69.3	**0.0004**
Total body water (%)	41.9 ± 4.9 35.5–60.3	53.6 ± 5.1 45.2–62.3	46.6 ± 7.2 37.8–64.8	47.3 ± 7.5 35.5–62.3	**0.0001**
BMR (kcal)	1381.9 ± 155.2 1199.0–1958.0	1667.7 ± 169.5 1436.0–2284.0	1438.9 ±158.9 1230.0–1794.0	1495.4 ± 202.5 1199.0–2284.0	**0.0001**
Bone mass (kg)	2.9 ± 0.5 1.9–3.6	2.9 ± 0.5 2.3–3.8	2.4 ± 0.3 2.0–3.0	2.8 ± 0.9 1.9–3.8	**0.0001**
Visceral tissue	1.8 ± 1.3 1.0–5.0	1.2 ± 0.5 1.0–3.0	1.1 ± 0.4 1.0–3.0	1.3 ± 0.9 1.0–5.0	**0.0001**

**Table 3:** Average evaluation of one’s own body based on the BES Body Evaluation Scale in aspects of items modifiable by diet and exercise and determining physical condition.

Assessment Aspect:	Control Group N = 50 (Points)	Volleyball N = 50 (Points)	Bodybuilding and Fitness N = 50 (Points)	Total N = 150 (Points)	*p*-Value
The aspect of ‘assessments of individual body parts’ (max 45 points)	27.4	29.4	32.1	29.7	**0.0022**
The aspect of ‘physical fitness of the body’ (max 50 points)	26.3	29.9	29.7	28.5	

**Table 4:** Evaluation of self-image of the Body Evaluation Scale BES in the studied groups of women (median). Bold values represent statistical significance.

Variable:	Control Group N = 50	Volleyball N = 50	Bodybuilding and Fitness N = 50	Total N = 150	*p*-Value
Appetite	3.0	3.5	3.0	3.0	0.9238
Physical fitness	3.0	4.0	3.0	3.0	**0.0001**
Reflex	3.0	4.0	3.0	3.0	0.8301
Muscle strength	2.0	3.0	3.0	3.0	**0.0008**
Waist	3.0	3.0	4.0	3.0	0.1183
Energy level	3.0	3.0	3.0	3.0	0.0665
Thighs	2.0	3.0	3.5	3.0	**0.0186**
Arms	3.0	3.0	3.0	3.0	0.0934
Body shape	2.0	3.0	3.0	3.0	**0.0063**
Physical coordination	3.0	3.0	4.0	3.0	0.0507
Buttocks	3.0	3.0	4.0	3.0	**0.0008**
Excitability	3.0	3.0	4.0	3.0	0.0530
Hips	3.0	2.0	4.0	3.0	**0.0003**
Legs	2.0	3.0	3.5	3.0	**0.0186**
Figure	3.0	3.0	3.0	3.0	0.5839
Abdomen	2.0	3.0	3.0	3.0	0.1190
Health	3.0	4.0	3.0	4.0	0.1650
Physical conditions	3.0	3.5	4.0	3.0	**0.0066**
Body mass	3.0	3.0	3.0	3.0	0.1205

**Table 5:** Assessment of self-esteem and self-confidence related to the appearance of one’s own body using a questionnaire in the opinion of female respondents (median).

Evaluation Aspect:	Control Group N = 50	Volleyball N = 50	Bodybuilding and Fitness N = 50	Total N = 150	*p*-Value
1. It disturbs me that I don’t look better.	3.0	3.0	2.5	3.0	**0.0213**
2. My self-esteem does not depend on whether I look better or worse.	3.0	3.0	2.0	3.0	**0.0007**
3. I am satisfied with my appearance.	3.0	3.0	2.0	3.0	0.8273
4. My self-esteem is affected by how attractive I find my face and its appearance	4.0	4.0	3.0	4.0	0.2096
5. I look better than the average person	3.0	3.0	3.0	3.0	0.2681
6. My self-esteem suffers whenever I think I don’t look good.	3.0	3.0	3.0	3.0	0.2258
7. I am happy that I look so good	3.0	2.0	3.0	3.0	0.2517
8. My self-esteem is not related to how I feel about my appearance	3.0	2.0	2.0	2.0	0.5414
9. Most people will probably consider me physically unattractive.	3.0	2.0	2.0	2.0	**0.0041**
10. When I think I look attractive. I feel better about myself.	4.0	4.0	4.0	4.0	0.0828
11. I would like the power to change my physical appearance.	4.0	4.0	3.0	4.0	**0.0015**
12. I would have had much more success on dates if I looked better.	3.0	3.0	2.0	3.0	**0.0002**

**Table 6:** Subjective directions of the desire to change their figure among the studied groups of women.

The Direction of Change:	Control Group N = 50	Volleyball N = 50	Bodybuilding and Fitness N = 50	Total N = 150	*p*-Value
	% (n)	% (n)	% (n)	% (n)	
**Reduce body fat**	40.0 (20)	50.0 (25)	24.0 (12)	57.0 (38)	**0.0075**
Reduce the amount of body fat and increase the amount of muscle tissue	40.0 (20)	10.0 (5)	6.0 (3)	36.0 (18)	
Increase the amount of body fat and increase the amount of muscle	12.0 (6)	0 (0)	0 (0)	6.0 (3)	
Increase the amount of muscle tissue	8.0 (4)	0 (0)	10.0 (5)	14.0 (7)	
Decrease amount of muscle tissue	0 (0)	4.0 (2)	0 (0)	1.3 (2)	

## Data Availability

Data are available on request due to restrictions of privacy or ethics.
